# SARS in Healthcare Facilities, Toronto and Taiwan

**DOI:** 10.3201/eid1005.030791

**Published:** 2004-05

**Authors:** L. Clifford McDonald, Andrew E. Simor, Ih-Jen Su, Susan Maloney, Marianna Ofner, Kow-Tong Chen, James F. Lando, Allison McGeer, Min-Ling Lee, Daniel B. Jernigan

**Affiliations:** * Centers for Disease Control and Prevention, Atlanta, Georgia, USA; † Sunnybrook and Women’s College Health Sciences Centre, Toronto, Ontario, Canada; ‡ Center for Disease Control, Taipei, Taiwan; § Health Canada, Ottawa, Ontario, Canada; ¶ Mount Sinai Hospital, Toronto, Ontario, Canada

**Keywords:** severe acute respiratory syndrome, infection control, delivery of healthcare

## Abstract

The healthcare setting was important in the early spread of severe acute respiratory syndrome (SARS) in both Toronto and Taiwan. Healthcare workers, patients, and visitors were at increased risk for infection. Nonetheless, the ability of individual SARS patients to transmit disease was quite variable. Unrecognized SARS case-patients were a primary source of transmission and early detection and intervention were important to limit spread. Strict adherence to infection control precautions was essential in containing outbreaks. In addition, grouping patients into cohorts and limiting access to SARS patients minimized exposure opportunities. Given the difficulty in implementing several of these measures, controls were frequently adapted to the acuity of SARS care and level of transmission within facilities. Although these conclusions are based only on a retrospective analysis of events, applying the experiences of Toronto and Taiwan to SARS preparedness planning efforts will likely minimize future transmission within healthcare facilities.

In March 2003, reports of healthcare workers with unexplained pneumonia in Vietnam initiated an international investigation of the infection that came to be known as severe acute respiratory syndrome (SARS) (*1*). The cause of SARS was later identified as a coronavirus, which was cultured from specimens provided by a healthcare worker who subsequently died of SARS (*2*). During the outbreak, transmission in hospitals and infection in healthcare workers persisted. In Toronto and Taiwan, nosocomial transmission played a substantial role in initiating and maintaining outbreaks of SARS. We summarize our experiences during these outbreaks to highlight key factors that can help healthcare and public health officials prevent nosocomial transmission of SARS. In addition, we offer conclusions based on an in-depth, retrospective analysis of the events as they unfolded in these two settings.

## High Risk of Transmission in Healthcare Workers, Patients, and Visitors

At the onset of the global outbreak, patients infected with SARS coronavirus (SARS-CoV) sought care at emergency departments for symptoms of what appeared to be common respiratory infections. During such encounters, minimal infection control measures were implemented since most known infections did not warrant them. However, in some circumstances, conditions were favorable for efficient transmission of SARS. Many exposed healthcare workers, patients, and visitors became infected and subsequently transmitted infection to others in their healthcare facilities. Nosocomial transmission was the primary accelerator of SARS infections, accounting for 72% of cases in Toronto (*3*) and 55% of probable cases in Taiwan (Table) (*4*).

**Table:** Characteristics of the SARS outbreak in the greater Toronto area and Taiwan, March–June 2003^a^

Characteristic	GTA, no. (%)	Taiwan, no. (%)^b^
Total cases	375	NA
Probable	247 (66)	668
Suspected	128 (34)	NA
Deaths	44 (12)	72 (11)
Healthcare related	271 (72)	370 (55)
Healthcare workers	164 (44)	120 (18)
Patients or visitors	107 (28)	256 (38)
Hospitals with hospitalized SARS patients	23	84
Hospitals with SARS transmission	10 (43)	8 (10)
Hospitals that closed wards or an emergency room	10 (43)	NA

^a^SARS, severe acute respiratory syndrome; GTA, greater Toronto area; NA, data not available ^b^Percentage expresses proportion of all probable SARS cases

**Figure 1:**
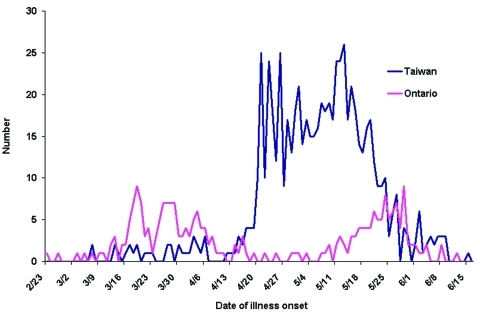
Number of probable cases of severe acute respiratory syndrome, by location and date of illness onset—Toronto and Taiwan, February 23–June 15, 2003.

In Toronto, the outbreak unfolded in two phases, both attributable to nosocomial transmission (Figure 1). The first phase resulted from a case of unrecognized SARS in an infected contact of a recent traveler to Hong Kong (*5*). The second phase resulted from unknown transmission of SARS among hospitalized patients during a period when healthcare workers were being instructed to wear personal protective equipment, including gowns, gloves, and masks (*6*). In Taiwan, the outbreak had two phases (Figure 1). The first phase consisted of sporadic SARS cases in travelers without nosocomial transmission (*7*). In the second phase, transmission at one municipal hospital ignited a number of subsequent nosocomial outbreaks when SARS patients were transferred to other facilities (*4*).

A number of factors may make nosocomial transmission a common mode of infection. Unlike many other viral respiratory diseases in which the concentration of virus is greatest on disease onset, the concentration of SARS-CoV in secretions appears to peak approximately 10 days after symptom onset (*8*) when a patient’s symptoms are often worsening and may require medical attention. Thus, patients may be most capable of transmitting the virus at the point when they encounter healthcare workers. In addition, transmission appears to be primarily through exposure to respiratory droplets and direct contact with patients and their contaminated environment (*5*,*9*). Healthcare workers and others in contact with SARS patients may be more likely to become infected, especially if exposed during aerosol-generating procedures (i.e., intubation, nebulizing medications). Finally, even after recognition of SARS, lapses in infection control measures may be responsible for infection in healthcare workers.

Whether SARS will occur again, and if so, whether the epidemiology will be similar to the outbreak in the spring of 2003 are not known. However, given the severity of illness seen in SARS patients and their eventual need for medical attention, healthcare workers and others in healthcare facilities are likely to remain at high risk if SARS reemerges.

## Variation in SARS Communicability

Over the course of the SARS outbreak, certain persons and settings were found to be more efficient at transmitting SARS-CoV infection than others. In Taiwan, after an initial period of apparent control of SARS by public health officials (*7*), exposures to an apparent “super-spreader” with SARS contributed to an explosion of infections at a municipal hospital in Taipei (*4*). An infected hospital laundry attendant continued working despite worsening symptoms of diarrhea and pneumonia. Between the onset of his illness and eventual recognition of SARS, exposures to the worker and to the hospital led to at least 137 probable cases, including 45 in healthcare workers. Similarly, a small number of persons also generated a large number of cases during the first phase of the Toronto outbreak when a cluster of healthcare workers were infected with SARS after the intubation of a severely ill SARS patient (*10*). Comparable transmission from one person to many was seen in Singapore as well (*11*).

In contrast, experiences with SARS in the United States and several other countries have not shown similar superspreading patients or events despite opportunities for transmission (*12*). The reasons for such variable communicability are uncertain but may be due to innate characteristics of infected patients (*13*), high virus concentrations in secretions during peak illness (*8*), or exposures to aerosol-generating procedures such as intubation or positive-pressure ventilation (*10*). Because these procedures are considered high risks for SARS transmission, the Centers for Disease Control and Prevention (CDC) has developed guidelines that emphasize use of PPE and, if needed, furlough for healthcare workers with unprotected exposure to these procedures (*14*).

## Transmission from Unrecognized Cases

On February 23, 2003, a 78-year-old Canadian woman returned from a visit to Hong Kong. While there, she had unknowingly been infected with SARS-CoV during her stay at a hotel in Kowloon (*5*). After returning to Toronto, the patient’s condition worsened, and she died at home. SARS developed in her son, and he was hospitalized with respiratory distress on March 7. Before his death on March 13, he infected two other patients and one healthcare worker, all of whom subsequently exposed others to the infection before SARS was eventually recognized. Infected visitors also contributed to transmission in the hospital. Ultimately, 128 cases were associated with this hospital outbreak, including 47 (37%) hospital staff and 36 (28%) patients and visitors (*5*). Many of these cases occurred early in the global outbreak and before SARS transmission was recognized in Canada. Once the disease was recognized, appropriate infection control practices were initiated so that by May 14, the World Health Organization advised that Toronto was no longer an “affected area” with the last locally acquired (recognized case having occurred on April 20, 2003.

After the first phase of SARS in Toronto, healthcare workers continued to use extensive personal protective equipment (e.g., routine contact precautions with an N95 or equivalent respirator). However, after a period with no apparent SARS transmission, public health officials relaxed the requirement for extensive PPE. Subsequently, a cluster of SARS cases occurred among healthcare workers, followed by the second phase of SARS transmission in Toronto (*6*). In retrospect, investigators determined that SARS-CoV transmission had continued undetected among patients. These unrecognized cases occurred later in the global outbreak and after recognition of SARS transmission in Canada.

The experiences from Taiwan and from both outbreak phases in Toronto underscore the difficulty in detecting SARS cases and the cascade of infections that can occur from even one unrecognized case among persons in the hospital. Symptoms of SARS are nonspecific and may represent infection due to a number of respiratory pathogens. Without rapid diagnostic tests, clinicians must rely for diagnosis on a patient’s history of travel, exposure to healthcare facilities, or contact with patients with suspicious cases of pneumonia. To prevent SARS transmission, all healthcare workers and visitors entering hospitals in Toronto and Taiwan were screened for symptoms or epidemiologic links to settings where transmission was known or suspected. Epidemiologic links are important discriminators for considering a diagnosis of SARS; however, before any global SARS activity and during periods of notable local transmission, these epidemiologic links may lose their discriminating ability. Ultimately, vigilant and intuitive clinicians may be the best means of recognizing cases of SARS.

### Minimizing Transmission through Early Detection and Intervention

Hospital emergency departments were important sites for SARS transmission during the early part of the outbreak in Toronto (*5*). In Taiwan, transmission in the emergency department occurred through unrecognized case-patients and during a period when infection control measures were weakened due to the rapid influx of SARS patients seeking evaluation. A number of administrative, engineering, and other controls were eventually implemented to minimize transmission of SARS in emergency departments in both Toronto and Taiwan. One important activity was “triage screening.” For this, a questionnaire was administered to entrants to identify SARS symptoms and exposures. Screening was accompanied by a temperature check, mandatory hand hygiene by the patient, and often by providing a surgical mask before admission to the hospital. These precautions were taken when the patient was first encountered by hospital staff.

**Figure 2:**
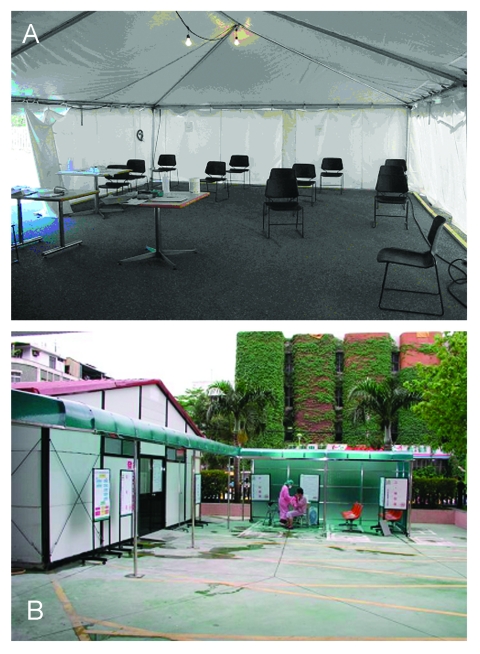
A, evaluation center for severe acute respiratory syndrome (SARS) in Toronto, demonstrating spatial separation of chairs in waiting area intended to reduce patient-to-patient transmission. B, evaluation center for SARS in Taiwan, demonstrating triage screening of a patient by a healthcare worker wearing personal protective equipment.

At the peak of the outbreaks in Toronto and Taiwan, healthcare providers and public health officials were faced with the possibility that any person coming to an emergency department with a febrile respiratory illness might have SARS and might transmit infection to other patients. In response, officials either constructed or retrofitted existing facilities to create SARS evaluation centers (i.e., “Fever Clinics”) (*13*). These units were designed to safely assess large numbers of people while minimizing the risk for SARS transmission, and in fact in both Toronto and Taiwan, no transmission was reported in these facilities. Staff and patients were grouped into cohorts, and a space of >l m was allocated between patients to make direct contact and droplet transmission less likely. Dedicated entrances and exits and clearly marked patient pathways were provided to segregate patients under evaluation. Provisions were made to ensure adequate ventilation and air exhaust to reduce the risk for droplet or airborne transmission. In Taiwan, temporary structures with high efficiency filtration were built (Figure 2). In Toronto, both tents and existing facilities were used.

### Strict Adherence to Infection Control Practices

Early in the global outbreak, SARS-CoV was frequently transmitted to healthcare workers. At that time, with no diagnostic assays or therapies, public health officials recommended personal protective equipment to prevent contact, droplet, and airborne transmission (*14*). In this situation, a large number of healthcare workers were required to wear gowns, gloves, N95 or higher respirators, and eye protection, often for hours. In the past, this level of protection had been recommended infrequently for those treating patients with such infections as active multidrug-resistant tuberculosis, suspected smallpox, or viral hemorrhagic fever (*15*). In Toronto and Taiwan, nurses, physicians, and housekeeping and other ancillary staff required rapid training to familiarize them with appropriate technique for the use of PPE. Additionally in Taiwan, training was needed for family and hired caretakers who provided a supportive healthcare function in hospitals in Taiwan (*4*).

To facilitate the complicated process of donning and removing personal protective equipment, officials developed videos, computer presentations, and posters to train and remind healthcare workers. In addition, changes in shift duration and rearrangement of nursing stations in SARS wards were needed to minimize lapses in infection control. However, as mentioned, even with fully protected healthcare workers, SARS transmission continued among patients, precipitating the second outbreak phase in Toronto. Glove use outside the immediate care environment was not recommended, but officials needed to emphasize the importance of removing gloves and washing hands after leaving SARS areas to prevent contaminating the environment or infecting patients.

Experiences in Toronto (*5*), Taiwan (*4*), and globally (*9*) indicate that the primary mode of SARS transmission is through direct contact and respiratory droplets. However, the cluster of SARS cases in Toronto healthcare workers after the intubation of a patient (*10*), as well as other reported superspreader events, suggest the possibility of limited airborne transmission under certain circumstances. Hand hygiene, one of the most important and simplest of interventions, was widely advocated both in the hospital and in public places. Surgical masks and respirators were recommended equipment for healthcare workers; however, use of masks and respirators in Taiwan became commonplace both in and outside the hospital. Inappropriate use of PPE caused shortages of supplies. In response, officials developed guidelines for respirator reuse and identified alternatives for equipment in short supply.

Infection control in Toronto and Taiwan became an essential public health activity, which required the implementation of precautions beyond most officials’ experience and expectations. Public health authorities took an active role in assessing the adequacy of control measures in hospitals and in investigating any potential transmission. Once widespread infection-control practices, along with other measures, were implemented, the number of new SARS cases declined.

### Minimizing Exposure Opportunities through Patient Isolation

Instituting recommended airborne transmission precautions for SARS patients required the use of airborne-infection isolation rooms, also known as “negative pressure” rooms. During early control of SARS in Taiwan, the small number of imported cases was adequately contained in these isolation rooms (*7*). After the rapid increase of cases, affected hospitals quickly exceeded their capacity to accommodate all patients in such isolation rooms. Two initiatives addressed the problem. First, government officials provided resources to build new airborn-infection isolation rooms at hospitals (*4*). Second, hospital officials grouped SARS patients in private rooms on dedicated, reengineered, SARS wards with modified ventilation systems that separated the ward airspace from the remainder of the hospital. Barriers of plastic sheeting and tape were constructed to limit access. When possible, SARS patients with pneumonia, who presented the highest risk for transmission, were placed in airborne-infection isolation rooms; other SARS patients were placed in private rooms on the SARS wards. Restricting SARS care to one unit or ward allowed the separation of contagious and noncontagious patients and limited the number of staff with potential exposures to SARS. Exposure opportunities were further minimized by maintaining a high staff-to-patient ratio and a high level of infection-control training on SARS wards.

In both Toronto and Taiwan, hospital officials restricted access to affected hospitals by limiting the number of entryways. Access stations were staffed with personnel to screen for fever, symptoms, or potential SARS exposures. Few visitors to SARS patients were allowed, and healthcare workers or visitors exposed to facilities where SARS transmission had occurred were not permitted to enter non-SARS areas. Hospitals with notable recent nosocomial transmission prevented visitors or nonessential staff from entering. Measures to limit access also included restrictions for transferring patients into or out of the hospital. If medically necessary, transfers were made after consultation with hospital and public health authorities.

Officials in Toronto and Taiwan considered designating a single facility to serve as a “SARS hospital” for their jurisdictions. However, implementing this policy was challenging. Facilities that were not seriously affected generally did not want to become the principal providers of SARS care because of concerns regarding liability, impact on finances, and negative public image. Ultimately, public health and healthcare officials chose to prepare and support many hospitals to provide care to SARS patients. This measure eliminated the need for a designated SARS hospital while maintaining a higher vigilance for SARS transmission at multiple facilities. In the second phase of the Toronto outbreak, four facilities where SARS patients were already residing were designated as SARS hospitals.

## Adapting SARS Control Measures to a Facility

Many infection control activities in Toronto and Taiwan were resource intensive and difficult to maintain for an extended period. To prevent unnecessary use of staff and materials, some measures were implemented only when transmission in the surrounding community or within the hospital reached a particular level. For example, using surgical masks throughout a hospital to contain infection in a healthcare worker or other person with symptoms was only implemented when transmission in the community was ongoing or recent transmission had occurred in the facility. Other functions, such as limiting access, restricting transfers, and performing surveillance for new-onset illness among healthcare workers, were initiated at different times in hospitals on the basis of hospital transmission or community transmission.

Closing an emergency department or hospital ward also was linked to the level of transmission within a hospital. Closings were necessary to prevent additional cases in a hospital where the risk for transmission was high or the source of transmission was unknown. However, given the substantial negative effect on hospital finances and healthcare access in a community, the decision to close a hospital to new admissions was made only in consultation with public health authorities.

## Conclusions

On July 5, 2003, the World Health Organization declared the world free of ongoing SARS transmission (*16*). However, the factors that led to the emergence of SARS are likely still in place, permitting the possibility that SARS will reemerge. If this happens, nosocomial transmission and cases among healthcare workers may also occur. Taking the experiences from Toronto and Taiwan and applying them to preparedness and prevention efforts likely will minimize SARS transmission in healthcare facilities.
